# Women’s experiences of female ejaculation and/or squirting: a Swedish cross-sectional study

**DOI:** 10.1093/sexmed/qfae074

**Published:** 2024-11-26

**Authors:** Jessica Påfs, Anna Wahlberg, Kerstin S Fugl-Meyer, Shirin Ziaei

**Affiliations:** Department of Social Work, University of Gothenburg, Gothenburg, 41123, Sweden; Department of Women's and Children's Health, Uppsala University, Uppsala, 75237, Sweden; Department of Neurobiology, Care Science and Society, Division of Family medicine and Primary care, Karolinska Institutet, Huddinge, Stockholm, 14152, Sweden; Department of Women's and Children's Health, Uppsala University, Uppsala, 75237, Sweden; Department of Food Studies, Nutrition and Dietetics, Uppsala University, Uppsala, 75237, Sweden

**Keywords:** female sexual pleasure, sexual response, orgasm, sexual functioning, quantitative research

## Abstract

**Background:**

Women’s experiences of the expulsion of fluids during sexual stimulation, commonly referred to as female ejaculation/squirting, are not well comprehended in the existing literature.

**Aim:**

To investigate women’s knowledge about and experiences of female ejaculation/squirting.

**Methods:**

Data from 1568 women (aged 18 to 69) were collected using a cross-sectional online-based questionnaire (in Swedish).

**Outcomes:**

The study focused on descriptive features of knowledge about, reactions to, occurrence of, and sensations around female ejaculation/squirting.

**Results:**

Among the participants, 58% had experienced ejaculation/squirting (significantly more often among non-heterosexuals). Among women without such experience, only one-third would like it to happen. Among women with such experience, ejaculation/squirting occurred consistently during sexual practice for a small percentage (7%) and on a few occasions for about half (52%). Despite most (77%) rating it as primarily a positive sensation, many reacted with shock/shame (28%) or thought they had urinated (26%) the first time it occurred. Many (61%) reported orgasm occurring close to, or simultaneously, with ejaculation/squirting, and these women were more likely to report it as a positive sensation (*P* < .001). Despite overall positive aspects, 58% had wanted to avoid it at some point, mainly because it got too wet or due to insecurities about the content of the fluid. Having wanted to avoid it at some point was more likely among those who sensed the fluid as being expelled from the urethra (*P* < .001) or whose partner reacted negatively to it (*P* < .001).

**Clinical Translation:**

This study contributes with a nuanced understanding of women’s experiences of ejaculation/squirting and related challenges.

**Strengths and Limitations:**

This study is the first to explore women’s desire for ejaculation/squirting, their initial reactions, and reasons for avoidance. There are limitations due to the nature of the data collection, such as missing data and potential overrepresentation of women who are informed and open-minded about ejaculation/squirting.

**Conclusion:**

Ejaculation/squirting is a common occurrence among women and, despite being perceived predominantly positively, particularly when linked with an orgasm, initial reactions, and avoidance underscore complexities related to excessive wetness and insecurities about the fluid’s content.

## Introduction

Female ejaculation and squirting are explained as the emission of fluid, other than vaginal lubrication, during sexual arousal or orgasm.^1–5^ There is still a discordancy on the exact definition, origin, and make-up of the fluid.^6^^,^^7^ Despite limited evidence, a semantic shift regarding female ejaculation/squirting was initiated in 2011. This was based on a single study, with just one study subject, who first expelled a clear, abundant fluid, referred to as “squirting,” with biochemical markers similar but not equivalent to urine, and understood to be expulsed from the bladder, and immediately after expelled a thicker, whitish fluid, referred to as “female ejaculation,” with biochemical markers comparable to some components of male semen and understood to be expulsed from the ﻿paraurethral glands^3^ (referred to by some as the female prostate).^8^ Due to the observed variations in biochemical markers, it was suggested that female ejaculation and squirting should be regarded as 2 distinct events. Despite being based on a study with just one study subject and the understanding that both can occur simultaneously, subsequent scholars have adopted this differentiation. A study from 2015 involving 7 participants investigated squirting and confirmed that the biochemical markers are similar to urine, containing varying concentrations of urea, creatinine, and uric acid, along with marginal components of ﻿prostate-specific antigen (PSA).^4^ The study did not control for fructose in the fluids, which has been pointed out as a marker distinguishing ejaculation from urine.^9^ Further studies with similar methodologies are warranted. Revisiting earlier literature becomes challenging due to the exclusive use of the term "female ejaculation," despite the likelihood that they were also portraying squirting. Similarly, it is questionable whether a distinction can be made; therefore, recent studies have also combined the terms,^10^ a decision we decided to follow in the current study.

Ejaculation/squirting is often referred to as a rarity^11^; however, a recent US-based study found the prevalence of squirting to be 41%,^12^ which is similar to previous studies on female ejaculation from the United States and Canada^13^ and Egypt.^14^ In Bullough et al.’s US-based study, women with a female partner were more likely to have had the experience.^15^

Women’s first discovery of ejaculation/squirting is relatively unmapped. For many women, it seems to occur unintentionally,^12^ and for some, a new partner was a trigger for the first occurrence.^16^ First-time reactions and whether or not women would like it to happen have not, to our knowledge, been assessed in quantitative studies. It has also been suggested that the concept of ejaculation/squirting is relatively unknown before a woman experiences it herself.^10^^,^^17^^,^^18^ Only one study, from 1990, has examined sources of information and identified traditional printed materials (books, journals, and magazines) as the predominant sources.^13^ Recently, pornography has been cited as an important source of information,^10^^,^^17^^,^^18^ but so far, no quantitative investigations have been undertaken.

The techniques used to ejaculate/squirt vary.^10^^,^^12^^,^^15–17^ Bullough et al. found that a combination of vaginal and clitoral stimulation was most common, while clitoral stimulation alone was enough for some. This led the authors to question the then-dominant, and still prevalent, idea that stimulation of the anterior vaginal wall, also named “the G-spot,” is necessary for a woman to ejaculate.^19^ This association originates from studies in the 1980s linking ejaculation with “the G-spot.”^20^ Perry and Whipple ^21^ termed it the Gräfenberg or G-spot, based on Gräfenberg’s ^22^ findings identifying an erotic zone along the anterior vaginal wall that, when stimulated, led to fluid expulsion from the urethra. While the term ``G-spot'' is misleading, as there is no specific anatomically confirmed spot responsible for all functions,^8^^,^^23^ stimulation of the anterior vaginal wall during sexual activity likely affects 5 distinct tissues: the erectile tissue of the clitoral crura or the clitoral bulbs, the glandular tissue surrounding the urethra, the urethra itself, and/or the anterior vaginal wall.^24–26^ The area has therefore been proposed to be termed the “G-zone”, clitoral complex or ﻿clitourethrovaginal (CUV) complex. ^8^^,^^24^^,^^25^^,^^27^ Recent research has confirmed that, in addition to using pressure inside the vaginal wall, and pressure concurrent outside/inside, women can also squirt by clitoral stimulation alone.^12^ However, the need for an enhanced comprehension of techniques has been expressed.^12^

Ejaculation/squirting does not necessarily occur in combination with orgasm, according to recent studies,^10^^,^^12^^,^^17^^,^^18^ while earlier studies often equated the definition of the event with orgasm.^13^^,^^15^^,^^16^ The moment at which women sense that the ejaculation/squirt is about to happen has not been investigated. Only a few scientific studies have explored women’s experiences of ejaculation/squirting.^10^^,^^12^^,^^13^^,^^15–18^ An international study by Wimpissinger and co-workers demonstrated ejaculation to be an enrichment of the sex lives of most women and their partners. In qualitative studies, women have expressed amazement and pride due to ejaculation/squirting, describing it as a unique, heightened sensation, or a visual representation of pleasure. However, women have also expressed shame due to its unfamiliarity, thinking it is uncommon, worrying about their partner’s reaction, the excessive amount of fluid, or the fear that it might contain urine.^10^^,^^17^^,^^18^

Given the scant, scattered, and partially outdated research on the topic, we identified specific knowledge gaps. These include: an updated understanding of the sources of information regarding female ejaculation/squirting; whether women want it to happen; how often it is part of the sexual response process; the reaction to it the first time it happened; the reaction of a partner; whether it relates to orgasm; whether women want to avoid it, and if so, why. Therefore, this study aims to answer the following research questions: What do women know about female ejaculation/squirting, and where have they learned about it? What experiences have women had with female ejaculation/squirting? How do women react to female ejaculation/squirting? What sensations do women report in relation to female ejaculation/squirting?

## Methods

### Study design and population

This descriptive cross-sectional study was conducted in Swedish using an online-based questionnaire (available between July and September 2019). Information about the study was disseminated on social media. People over the age of 18 and able to understand Swedish were eligible to participate. The present study did not aim to measure prevalence. Acknowledging and inspired by previous studies on the topic,^13^^,^^15^^,^^16^ and by information and experiences of preliminary findings from a qualitative study on the topic done by the first author,^10^ the questionnaire was created by the first and second authors and reviewed by the third author and an external researcher within the field of sexology. It was pilot tested and small revisions were then made prior to starting data collection. The Swedish word “fontänorgasm” (directly translated as “fountain orgasm”) was used and described as being ejaculation of either a milky fluid and/or a larger amount of transparent fluid, expelled before/in connection with/after orgasm, or without orgasm, in line with definitions from previous studies.^3^^,^^4^ The current study uses the terminology ejaculation/squirting to encompass the sought after experiences of both female ejaculation and squirting. The full questionnaire consisted of 64 nominal scale questions; the average time for completion was 15 (+/−5) minutes; 15 single-select multiple-choice questions covered demographic and social characteristics, 28 questions covered genital modifications and practices (not included in this study), and 21 questions covered women’s knowledge and experiences of ejaculation/squirting. Of the latter, 11 questions were single-select multiple-choice questions, 3 were multi-select multiple-choice questions, and 7 were open-ended questions (answers coded manually). Only those who had experienced ejaculation/squirting received the questions about their experiences of, reactions to, and sensations in relation to ejaculation/squirting. The open-ended questions included how they got to know about ejaculation/squirting, the type of stimulation used to ejaculate/squirt, their reaction the first time it happened, sensations during squirting, and sex-partners’ reactions. Those women who had not experienced ejaculation/squirting were only asked whether they would like to experience it or not. See Appendix 1 for the questions from the questionnaire analyzed in this article. Only respondents who identified themselves as women, and had a vagina, were included (*n* = 1568 (92.9%)).

### Ethics

The project was approved by the Swedish Ethical Review Authority (Dnr. 2019-01640). Participants received study details, emphasizing anonymity and voluntary participation; to access the questionnaire, confirmation that one had read and agreed to the information was required by checking a box.

### Statistical analysis

Data is presented as frequencies and percentages for the categorical variables and mean and standard deviation (sd) for the continuous variables. Chi-square tests were used to evaluate: the associations between experiences of ejaculation/squirting (yes/no) and sexual orientation (hetero/non-hetero sexual); timing of orgasm (close to or simultaneously with ejaculation/squirting versus not close or not related to ejaculation/squirting) and overall experience of ejaculation/squirting (positive vs negative or both negative and positive); the sensation of where the ejaculation/squirt is being expelled from (urethra vs vagina or other areas like glands around vagina or urethra) and whether or not they have tried to avoid it; their partner’s reaction (positive or neutral/indifferent/did not notice vs negative/both positive and negative) and whether or not the respondents have tried to avoid it. Pearson’s chi-square (χ^2^), degree of freedom (df), and *P*-values are reported. The criterion for statistical significance was set to 0.05. IBM SPSS Statistics, version 24 (IBM, Armonk, NY) was used.

## Results

As shown in Table 1, participants were mainly aged 26–40 years old (52%), with a mean age of 30 (±9.2), the majority were born in Sweden (93%), had higher education (71%), and had a heterosexual orientation (72%). About two-thirds were married/in a relationship and the same proportion had not given birth.

**Table 1:** Background characteristics of all women answering the questionnaire (*n* = 1568).

	*n/N*	%
Age		
18–25 years	542/1568	34.6
26–40 years	819/1568	52.2
41–69 years	207/1568	13.2
Country of birth		
Sweden	1439/1545	93.1
Others	106/1545	6.9
Educational level		
Primary/secondary school	446/1522	29.3
University/College	1076/1522	70.7
Marital status		
Married	212/1558	13.6
In a relationship	820/1558	52.6
Single	480/1558	30.8
Others^a^	46/1558	3.0
Experience of childbirth		
No	1092/1566	69.7
Yes, vaginal birth	376/1566	24.0
Yes, caesarean section	49/1566	3.1
Yes, both vaginal and caesarean section	49/1566	3.1
Sexual orientation		
Heterosexual	1119/1558	71.8
Pan or bisexual	338/1558	21.7
Homosexual	24/1558	1.5
Others^b^	77/1558	4.9

Total sample size varies due to missing data.

a Includes those who did not want to define their marital status.

b Includes those who did not want to define their sexual orientation.

Table 2 displays knowledge and personal experience. Almost all the participants were familiar with the concept of female ejaculation/squirting, and had first heard about it at an average age of 19 (±6.3) years. Their main sources of information were friends or partners (31%), followed by the media (29%), and pornography (16%), while only a few had received such information via education at school (4%), or through personal experience (4%).

**Table 2:** Knowledge and personal experience of female ejaculation/squirting among all participants (*n* = 1568).

	*n/N*	%
Familiar with the concept of female ejaculation/squirting		
Yes	1416/1425	99.4
Age when first heard about it (Mean ± SD)	18.99 ± 6.26	
Knowledge of ejaculation/squirting from^a^		
Friends/partner	394/1279	30.8
Media	376/1279	29.4
Pornography	209/1279	16.3
Education at school/lectures/workshops	54/1279	4.2
Personal experience	49/1279	3.8
Do not remember	197/1279	15.4
Personal experience of ejaculation/squirting		
Yes	728/1250	58.2
No	449/1250	35.9
Not sure	73/1250	5.8
No experience—but would like it to happen^b^		
Yes, very much	130/446	29.1
Maybe	165/446	37.0
It makes no difference	102/446	22.9
No	49/446	11.0

Total sample size varies due to missing data.

a Open-ended question

b Asked only of women who had not experienced ejaculation/squirting but would like it to happen (*n* = 449, missing *n* = 3).

More than half of the women (58%) had personal experience of ejaculation/squirting, and some (6%) were unsure if it had ever occurred. Of those with no personal experience of ejaculation/squirting, 29% wished to experience it, while others answered “maybe” (37%), were indifferent (23%), or replied “no” (11%). Women with non-heterosexual orientations were more likely to have experienced ejaculation/squirting than women with heterosexual orientation (63.3% vs 52.1%; χ^2^ = 14.169, *P* < 0.001) (Supplementary Table A).

In Table 3, the occurrence and reactions among women who had ever experienced ejaculation/squirting are reported. The data demonstrates that most participants were together with someone the first time ejaculation/squirting occurred, and that this was their typical experience. Ejaculation/squirting was reported to occur on a few occasions (52%) or sometimes (23%) during sexual activity for the majority, and always (7%) or about half the time (16%) for a smaller proportion. Vaginal stimulation, or a combination of vaginal/clitoral stimulation, was mostly used to ejaculate/squirt. The most common reaction the first time ejaculation/squirting occurred was a feeling of shock and shame (28%), and many thought they had urinated (25%). However, almost as many (27%) described it in positive terms. Reactions from sexual partners had mainly been positive (86%).

**Table 3:** Occurrence and reactions to female ejaculation/squirting among women who have ever experienced ejaculation/squirting (*n* = 728).

	*n/N*	%
First time ejaculation/squirting occurred		
Alone	233/708	32.9
Together with someone	475/708	67.1
Usually experience ejaculation/squirting		
Alone	174/687	25.3
Together with someone	513/687	74.7
Ejaculation/squirting included in sexual practice		
Always	47/648	7.3
About half the time	104/648	16.0
Sometimes	146/648	22.5
On a few occasions	337/648	52.0
Do not know	14/648	2.2
Type of stimulation used to ejaculate/squirt^a^		
Vaginal	224/624	35.9
Clitoral	87/624	13.9
Both vaginal and clitoral	240/624	38.5
Others^b^	71/624	11.4
Own reactions the first time it happened^a^		
Positive	179/665	26.9
Disappointed	101/665	15.2
Thought it was urine	169/665	25.4
Shocked/ashamed	186/665	28.0
Mixed (both positive and negative)	30/665	4.5
Partner’s reaction^a^		
Positive	497/581	85.5
Neutral/indifferent/did not notice	58/581	10.0
Negative	16/581	2.8
Mixed (both positive and negative)	10/581	1.7

Total sample size varies due to missing data.

a Open-ended question.

b Answers were: Stimulation around the urethra/anus/nothing specific/everything works/mood/dependent on partner/being alone.

Table 4 covers the sensations accompanying ejaculation/squirting among women who have ever experienced it. Primary sensations related to ejaculation/squirting were mostly positive (77%). Many (61%) reported orgasm to occur close to/simultaneously with ejaculation/squirting, while for some (19%) it was never/rarely connected. Quite a few (16%) chose the option “other” and most commonly described that it differed from time to time. Women were more likely to report ejaculation/squirting as positive if it occurred close to/simultaneously with an orgasm (83.3% vs 66.8%; χ^2^ = 24.739, *P* < 0.001) (Supplementary Table B). Women sensed the ejaculation/squirt mostly before (38%), or at the moment of (35%) the release. Most women sensed the ejaculate/squirt being expelled from the vagina (35%), or the urethra (26%).

**Table 4:** Sensations of female ejaculation/squirting among women who have ever experienced ejaculation/squirting (*n* = 728).

	*n/N*	%
Primary sensations around ejaculation/squirting		
Positive	540/700	77.1
Negative	54/700	7.7
Both positive and negative	106/700	15.1
Orgasm in relation to ejaculation/squirting		
Close to or simultaneously	420/686	61.2
Before or after	26/686	3.8
Never or rarely connected	131/686	19.1
Others^a^	109/686	15.9
Sensation of ejaculation/squirt occurring		
Beforehand	258/687	37.6
As it occurs	241/687	35.1
Afterwards	131/687	19.1
Others^b^	57/687	8.3
Sensation of the ejaculation/squirt being expelled from		
Vagina	242/686	35.3
Urethra	180/686	26.2
Others^c^	42/686	6.1
Do not know	222/686	32.4
Overall sensation of ejaculation/squirting^d^		
Pride/pleasurable	563/685	82.2
Ashamed/inconvenienced	53/685	7.7
Mixed sensations^e^	229/685	33.4
Indifferent	62/685	9.1
Ever wanted to avoid ejaculation/squirting	391/673	58.1
Reasons for wanting to avoid ejaculation/squirting^d^^,^^f^		
Gets too wet	308/391	78.8
Insecurity about content of fluid	126/391	32.2
Embarrassing	87/391	22.3
Believe it to be urine	75/391	19.2
Does not feel good	31/391	7.9
Sex partner does not like it	23/391	5.9

Total sample size varies due to missing data.

a Differed from time to time/dependent on the technique used.

b Differed from time to time.

c e.g. differed from time to time/both vagina and urethra/glans surrounding vagina/urethra.

d Multi-select multiple-choice question.

e Like it but not its wetness.

f Asked only of women who had tried to avoid ejaculation/squirting (*n* = 391).

The overall sensation of ejaculation/squirting (multiple-choice question) was reported by a large majority (82%) as pride/pleasurable, while a few (8%) felt ashamed/inconvenienced. One third (33%) reported mixed sensations of liking it but not liking the wetness. When asked if they had ever wanted to avoid ejaculation/squirting, 58% answered yes, mostly because of it getting too wet (79%), insecurity about the content of the fluid (32%), or embarrassment (22%). Women who sensed that the ejaculate/squirt was expelled from the urethra were significantly more likely to have tried to avoid ejaculation/squirting than others (74% vs 52.3%; χ^2^ = 25.181, *P* < 0.001) (Supplementary Table C). Women were also more likely to avoid ejaculation/squirting if their partner(s) had reacted negatively or both positively and negatively than those whose partners reacted positively or neutrally/indifferently/did not notice (92% vs 57.1%; χ^2^ = 12.003, *P* < 0.001) (Supplementary Table D). Finally, when the women were asked if they wanted to know about the content of the fluid and/or from where it is expelled, 43% said yes (results not shown in the table).

## Discussion

To the best of our knowledge, this study is the first to investigate whether women wish ejaculation/squirting to happen, their first-time reaction to it, and reasons for avoidance.

Female ejaculation/squirting was a familiar concept among the women in this study, yet more information about it was also warranted. The main sources of information being friends or partners, or the media, provides an updated understanding of where this knowledge is retrained. It was rare to have received this information during school sex education, while one common source of information was pornography.^18^ Pornography has increasingly featured depictions of female ejaculation and squirting, often portrayed as a visual demonstration of the female orgasm, analogous to the male ``money shot,'' challenging the perceived lack of a clear visual signifier for female climax.^19^^,^^28–30^ This representation in pornography may create unrealistic expectations that a complete orgasm includes ejaculation/squirting; however, it also contributes to normalizing the existence of ejaculation/squirting.^10^^,^^18^ Previous studies also suggest that the earlier women become aware of the existence of female ejaculation/squirting, the greater the likelihood that they will experience it themselves,^31^ further emphasizing the importance of including information about ejaculation/squirting in school sex education. Interestingly, in contrast to the idea of ejaculation/squirting being a sought-after experience, the results of this study indicate that, for many, it makes no difference or is an experience they are not interested in. The reason behind a wish to not experience it calls for further studies.

The present study did not aim to measure prevalence; however, the occurrence of 54% is in line with previous studies.^12^^,^^15^ It is worth noticing that 6% of the women in our study were unsure of whether or not they had experienced ejaculation/squirting, and among those with the experience, one-fifth sensed the ejaculation/squirting only after it occurred. In agreement with others,^10^^,^^17^^,^^18^ these findings indicate that it may not be obvious to the woman whether she has ejaculated/squirted. Furthermore, as demonstrated in the present study and others,^12^^,^^15^^,^^16^ ejaculation/squirting is part of a response to sexual stimulation or sexual arousal that may occur sometimes or on a few occasions, rather than consistently.

Women who had a non-heterosexual orientation were more likely to have experienced ejaculation/squirting, in line with Bullough et al.’s findings.^15^ This might suggest that women who engage in sexual activities outside the heterosexual script are more likely to experience ejaculation/squirting, potentially implying that the occurrence of ejaculation/squirting has a similar pattern to the prevalence of orgasm in heterosexual practices, also coined as the “orgasm-gap” or “pleasure-gap.”^32^ This would be an interesting hypothesis to explore further. Also, the present study suggests that clitoral stimulation alone can lead to ejaculation/squirting, in line with Hensel et al.’s recent study.^10^ This challenges the prevalent idea that stimulation of the anterior wall of the vagina is necessary to ejaculate/squirt.^19^^,^^20^

The first time that ejaculation/squirting occurred, most women reacted with shock and/or shame, or thought they had urinated. This sheds light on the potential lack of awareness or misconceptions about this response to sexual arousal, or expecting other sensations connected to it, in line with previous qualitative studies.^10^^,^^17^^,^^18^ Furthermore, the findings confirm that ejaculation/squirting may not be correlated with orgasm, underscoring the independence of the spurt of fluid and the occurrence of orgasm.

Half of the respondents had wanted to avoid ejaculating/squirting at some point, indicating that there was a certain discomfort around this aspect of sexual response. The reasons given were: (i) getting too wet, (ii) insecurity about the content of the fluid, (iii) embarrassment, and (iv) believing it to be urine. These highlight the concerns about wetness, an aspect suggested to contribute to women’s inability to relax and enjoy sexual practices.^33^ In this study, one-third of the women were unsure where the fluid was expelled from, with about one-third each believing it was ejected from either the vagina or the urethra. Similar results have been found in previous research.^13^^,^^16^ Women who sensed the fluid as being expelled from the urethra were more likely to have tried to avoid it. This is in line with the work of Darling and co-workers,^31^ who found that women were more likely to hold back an orgasm due to fear of urinating. This is also confirmed in a qualitative study illustrating how women dealt with the aspect that the fluid might be urine, despite their own convinced interpretation that the fluid is different in its nature.^10^ The latest research on the main components of the fluid^4^^,^^34^ has sparked media headlines suggesting that squirting is merely urine.^35^ With media being one of the most common sources of information, it can be suggested that this contributes to shame around this sexual response, as also discussed elsewhere.^10^^,^^18^ Further scientific evidence is imperative to elucidate the mechanisms underlying female ejaculation/squirting and the components of the fluid.

A negative reaction from a partner may make women more likely to try to avoid ejaculation/squirting. Even though quite a few women had experienced such reactions, this is a higher percentage than reported in previous studies.^16^ Not surprisingly, partners’ reactions play an important role in how women respond to their ejaculation/squirting. This is supported by qualitative studies in which women’s experiences, particularly the first time it happened, were influenced by their partner’s reaction.^10^^,^^17^^,^^18^ This was also found to be a reason why women express concerns about squirting.^12^

### Strengths and limitations

One strength of the present study is the large sample size. However, as the questionnaire was available and disseminated online, the participants may include an overrepresentation of women who are more informed and/or open-minded about sexuality and sexual practices. Since the study relied on self-reported data, the possibility of inaccurate responses cannot be ruled out. No attempt was made to ensure the representativity of the general population. Additionally, due to the cross-sectional nature of the study, causal inference cannot be established, which is another limiting factor. The questionnaire used in this study was carefully designed and pilot-tested; however, formal validation was not conducted within the scope of this research, which poses a limitation and may affect the generalizability of the findings. While this study contributes with more understanding, clinical examinations to further understand ejaculation/squirting, and whether they can be seen as 2 separate events, are warranted.

## Conclusion

This study confirms that women are familiar with the concept of ejaculation/squirting and that it occurs quite commonly, yet highlights varied experiences. While women generally perceived it as a positive experience, initial reactions and reasons for wanting to avoid it highlight challenges related to excessive wetness and insecurities about the fluid's content, underscoring the complexities of ejaculation/squirting within the sexual response process. Ejaculation/squirting can occur close to, or simultaneously with, an orgasm, although this is not always the case; however, it may be more likely to yield positive sensations when connected with an orgasm.

## Supplementary Material

Appendix_I_questionnaire_qfae074

Supplementary_Tables_female_ejaculation_qfae074
