# Supplementary material for: Women’s experiences of female ejaculation and/or squirting: a Swedish cross-sectional study
Source: Sex Med. 2024 Nov 26;12(5):qfae074. doi: 10.1093/sexmed/qfae074 (PMC11596687; doi:10.1093/sexmed/qfae074)
Supplement: Appendix_I_questionnaire_qfae074 [file appendix_i_questionnaire_qfae074.doc]

**Appendix I: Questionnaire**

Below are the questions used for the analysis in the article.

Background characteristics

**How old are you?** (open response option)

**Do you identify as:** Woman / Man / Prefer not to answer / Other: (please specify)

**What genitalia do you have?** Vagina / Penis / Prefer not to answer / Other (please specify):

**Country of birth** (list of all countries)

**Educational level:** Primary and Lower Secondary School (Grades 1-6) **/ Upper Secondary School (Grades 7-9) / High School (Grades 10-12) / Vocational Diploma / Bachelor's Degree / Master's Degree / Doctorate / Other:**

**Marital status:** Married **/** In a relationship **/** Single **/** Prefer not to answer **/** Other

**Have you given birth to one or more children?** No / Yes, vaginal birth / Yes, caesarean section / Yes, both vaginal and caesarean section **/** Prefer not to answer

**How do you define your sexual orientation? Select those that apply to you:** Heterosexual / Bisexual / Pansexual / Homosexual / Queer / Prefer not to categorize myself / Don't know / Prefer not to answer / Other:

**Prior to the next section, the following information was provided:**

**Sexual Pleasure and Female Ejaculation/Squirting*.** The following questions relate to female ejaculation/squirting* experienced by women/vulva owners. Ejaculation/squirting includes both the ejaculation of a milky fluid and/or a larger amount of clear fluid, regardless of whether the fluid is released before/during/after orgasm or without orgasm.

**Are you familiar with the concept of female ejaculation/squirting*?** Yes/No

**How old were you when you first heard about it?** (nominal scale)

**Describe where/how you got to know about female ejaculation/squirting*:** (open-ended response option)

**Do you have personal experiences of female ejaculation/squirting*?** Yes, I have experienced it / I am not sure it has happened to me / No **/** Do not want to answer

*If answered yes or not sure, the below questions were provided, otherwise the person received this follow up question*: **Would you like it to happen?** Yes, very much / Maybe / It makes no difference / No

**The first time ejaculation/squirting occurred, did it happen alone or together with someone?** Alone / Together with someone

**Do you usually experience ejaculation/squirting alone or when you are together with someone?** Alone / Together with someone

**Around how often is female ejaculation/squirting included in your sexual practice?** Always / About half the time **/** Sometimes **/** On a few occasions **/** Don’t know

**What type of stimulation do you use to ejaculate/squirt?** (open-ended response option)

**How did you react the first time it happened? Describe as thoroughly as possible:** (open-ended response option)

**How has the person you have had sex with usually reacted when you ejaculate/squirt*?** (open-ended response option)

**What are your primary sensations around ejaculation/squirting*:** Positive **/** Negative **/** Both positive and negative

**Do you experience orgasm in relation to ejaculation/squirting* (release of the fluid)?** I have an orgasm long before the fluid comes / I have an orgasm just before the fluid comes / I have an orgasm at the same time as the fluid comes / I have an orgasm just after the fluid comes / I have an orgasm long after the fluid comes / I rarely have an orgasm in connection with the fluid coming / I have never had an orgasm in connection with the fluid coming / I don't know / Other (please describe).

**When you ejaculate/squirt, do you sense it beforehand, as it occurs or afterwards?** Beforehand / As it occurs / Afterwards / Other? (Please specify)

**Where do you sense the fluid is being expelled from?** From the vagina / From the urethra / Don’t know / Other (please specify)

**What are your overall sensations regarding ejaculation/squirting*?** Check all the options that apply (Multi-select multiple-choice question): I find it cool / I feel proud over what my body can do / I find it pleasurable / I like that it gets sticky and/or wet / I feel ashamed / I do not like it and dislike that it gets sticky and/or wet / I like it but not that it gets sticky and/or wet / I feel indifferent

**Has it ever happened that you wanted to avoid to ejaculate/squirt*, why?** Check all the options that apply (Multi-select multiple-choice question): I think it gets too wet / I feel insecurities about content of fluid / I find it embarrassing / I believe it to be urine / It does not feel good / Sex partner does not like it

* The Swedish word “fontänorgasm” was used
