# Supplementary material for: Women’s experiences of female ejaculation and/or squirting: a Swedish cross-sectional study
Source: Sex Med. 2024 Nov 26;12(5):qfae074. doi: 10.1093/sexmed/qfae074 (PMC11596687; doi:10.1093/sexmed/qfae074)
Supplement: Supplementary_Tables_female_ejaculation_qfae074 [file supplementary_tables_female_ejaculation_qfae074.doc]

**Supplementary Table A**. Bivariate analysis of women’s sexual orientation and experience of female ejaculation/squirting among all women answering the questionnaire (n=1568)^1^

|  | *Experience of ejaculation/squirting* | | P value^2^ |
| --- | --- | --- | --- |
|  | No experience | experienced | <0.001 |
| *Sexual orientation* | n/N (%) | n/N (%) |
| Heterosexual | 452/943 (47.9) | 491/943 (52.1) |
| Non-heterosexual | 143/390 (36.7) | 247/390 (63.3) |

^1^ Total sample size reduced due to the missing values

^2^ P value was calculated using a chi-square test

**Supplementary Table B**. Bivariate analysis of the timing of orgasm and the sensations towards female ejaculation/squirting among women who have ever experienced ejaculation/squirting (n = 728)^1^

|  | *Female’s sensations towards ejaculation/squirting* | | P value^2^ |
| --- | --- | --- | --- |
|  | Positive | Negative/both positive and negative | <0.001 |
| *Timing of orgasm* | n/N (%) | n/N (%) |
| Close to/simultaneously with ejaculation/squirting | 349/419 (83.3) | 70/419 (16.7) |
| Not close to/not related to ejaculation/squirting | 175/262 (66.8) | 87/262 (33.2) |

^1^ Total sample size reduced due to the missing values

^2^ P value was calculated using a chi-square test

**Supplementary Table C**. Bivariate analysis of the sensations towards where the ejaculation/squirting expelled from and if the women tried to avoid female ejaculation/squirting (n = 728) ^1^

|  | *Tried to avoid ejaculation/squirting* | | P value^2^ |
| --- | --- | --- | --- |
|  | Yes | No | <0.001 |
| *Sensation of the ejaculation/squirt being expelled from* | n/N (%) | n/N (%) |
| Urethra | 131/177 (74.0) | 46/177 (26.0) |
| Vagina/other | 259/495 (52.3) | 236/495 (47.7) |

^1^ Total sample size reduced due to the missing values

^2^ P value was calculated using a chi-square test

**Supplementary Table D**. Bivariate analysis of partner’s reaction to women’s ejaculation/squirting and if the women tried to avoid female ejaculation/squirting (n = 728) ^1^

|  | *Tried to avoid ejaculation/squirting* | | P value^2^ |
| --- | --- | --- | --- |
|  | Yes | No | <0.001 |
| *Partner's reactions to women's ejaculation/squirting* | n/N (%) | n/N (%) |
| Positive or neutral/indifferent/did not notice | 306/536 (57.1) | 230/536 (42.9) |
| Negative/both positive and negative | 23/25 (92.0) | 2/25 (8.0) |

^1^ Total sample size reduced due to the missing values

^2^ P value was calculated using a chi-square test
